# Progeny array analysis to estimate outcrossing rates, inbreeding coefficients, and inbreeding depression among native, naturalized, and invasive populations of *Mimulus guttatus* (Phrymaceae)

**DOI:** 10.3389/fpls.2024.1411868

**Published:** 2024-11-21

**Authors:** Michele R. Dudash, Jason A. Berg, Elizabeth A. Zimmer

**Affiliations:** ^1^ Department of Natural Resource Management, South Dakota State University, Brookings, SD, United States; ^2^ Department of Biological Sciences, University of Maryland, College Park, MD, United States; ^3^ Department of Botany and Laboratories of Analytical Biology, National Museum of Natural History, Smithsonian Institution, Washington, DC, United States

**Keywords:** colonization, mating system, sexual reproduction, asexual reproduction, non-native populations

## Abstract

The mating system of non-native plant populations plays a role in determining the colonizing success following introduction into locations outside of the native distribution. For plant species capable of mixed-mating, both selfing and outcrossing can be advantageous and promote the establishment, persistence, and spread of newly arrived populations. To investigate how mating systems may contribute to the invasion process we estimated mating system parameters in perennial populations of the model plant species, *Mimulus guttatus* from its native range (West coast USA), non-native populations that are established but have not become invasive (East coast USA, >50 years), and populations in invasive regions (UK >200 years). Studies that include mating system data across the continuum of the invasion process are rare, thus here we utilize molecular markers to estimate outcrossing rates (t), inbreeding coefficients (F), and inbreeding depression in native, naturalized, and invasive populations. Overall, we found support for the persistence of mixed-mating across populations, variability in the relationship between outcrossing rates and inbreeding depression across populations, and evidence for the bridgehead process, where non-native populations may be the sources for the further establishment or reinforcement of additional non-native populations. The methodology we deployed had its own assumptions and sampling design constraints, that contributed to the variation in the parameter estimates. All *M. guttatus* populations likely rely on selfing through both within clone, and within flower and plant mating in addition to vegetative propagation. The results underscore the importance of introduction history in determining the role of both sexual and asexual reproduction in the successful establishment of a plant species outside its native range.

## Introduction

In hermaphroditic plants, self-compatibility is common, and populations of the same species can vary substantially in the degree to which they rely on outcrossing (Goodwillie et al., 2005; Whitehead et al., 2018). In native early-successional species (i.e. “weedy” species) or non-native plant species, outcrossing can provide a selective advantage by increasing the standing genetic variation required by populations to adapt to novel environmental conditions through admixture and recombination (Sax et al., 2007; Catford et al., 2009) which can also be beneficial in many management plans to address a lack of genetic variation in species of concern (Ralls et al., 2018; Rodger et al., 2024). However, we know much less about the mating system or how non-native naturalized or invasive populations persist outside their range and the factors that impact their expansion. From the literature we expect non-native plant species to begin as small populations during colonization owing to limited habitat, lack of pollinators and dispersal limitations inherent to sedentary life forms (Baker, 1967; Kolar and Lodge, 2001). In cases like these, a uniparental mode of reproduction, such as clonal propagation or self-fertilization (both within flower and geitonogamy within ramets), may be more selectively advantageous for population persistence (Vallejo-Marin and O'Brien, 2007; Pannell et al., 2015).

Given the adaptive advantages of both selfing and outcrossing are dependent on demographic and stochastic factors, many empirical and theoretical studies have led to the development of this field over time. One scenario is where complete outcrossing (*t* = 1) or complete selfing (*t* = 0) are alternative stable states (Lande and Schemske, 1985; Schemske and Lande, 1985) and propose that when intermediate outcrossing rates (0.2 < *t* < 0.8) are found, this “mixed mating” strategy, is simply a transitional state toward complete selfing or outcrossing. However, another scenario is also supported through theoretical (Latta and Ritland, 1994) and empirical studies and suggest that mixed-mating is an alternative stable state (Goodwillie et al., 2005; Jarne and Auld, 2006; Winn et al., 2011; Dart et al., 2012; Zhang et al., 2024). Both scenarios are based on the 3/2 transmission advantage following selfing compared to outcrossing (Fisher, 1941) and suggest that relaxation of certain selective forces can result in a stable mixed-mating strategy or complete selfing depending on the magnitude of inbreeding depression. It is during the early stages of colonization and establishment in part owing to lag effects in population expansion (Morgan et al., 2024) that the trajectory towards one of these three mating system strategies initially emerges although it could change over time following establishment.

The strongest selective force facilitating the transition from selfing to outcrossing is inbreeding depression, the decrease in fitness of progeny produced by matings between related individuals, i.e., selfing, relative to progeny produced from matings between unrelated individuals, i.e., outcrossing (e.g., Lande and Schemske, 1985; Charlesworth and Charlesworth, 1987; Dudash, 1990; Zhang et al., 2024; Rodger et al., 2024). Fitness is defined as both the quantity and quality of the progeny produced and can include various life history stages (ex., seed set, seed viability, germination, vegetative and floral size) depending on the study conducted. While inbreeding depression is likely the primary selective force, studies have suggested that the effects of inbreeding depression can be mitigated over time as the deleterious recessive alleles responsible for the decrease in fitness may be purged from the population (e.g., Hedrick, 1994; Dudash and Carr, 1998; Byers and Waller, 1999; Dussex et al., 2023).

Colonization success and its relationship to mating system has long been suggested and coined as “Baker’s Law” (Baker, 1955), where self-compatible species should be more successful colonizers following long-distance dispersal compared to obligate outcrossing species, in part because the former would need only one individual to establish a naturalized population (Baker, 1967; Stebbins, 1957; Kolar and Lodge, 2001). However, empirical evidence has both supported and contradicted this theory (Pannell and Barrett, 1998; Randle et al., 2009; Cheptou, 2012; Pannell et al., 2015) and have further highlighted a role for unisexual reproduction in colonization success (Pannell et al., 2015; Pantoja et al., 2017), thus there are likely multiple paths for establishment of plant populations outside of their native range. Furthermore, naturalization of a non-native plant species is often considered an intermediate critical stage prior to a population becoming invasive, representing a lag phase of slow population growth as it deals with deficiencies inherent to a novel population’s demographics or to maladaptation (Aikio et al., 2010; Murren et al., 2009; Richardson and Pyšek, 2006). Thus, various factors ultimately determine whether a non-native population goes extinct, remains cryptic and benign, or alternatively adapts and spreads into new locations (Catford et al., 2009; Richardson and Rejmánek, 2011).

The mode of reproduction, asexual versus sexual with the latter dependent on the mating system is key to how it may affect invasion success by influencing population size, dispersal, and genetic structure of introduced plant populations (Kinlan and Hastings, 2005; Bazin et al., 2014; Zimmer et al., 2022). Invasive plant species are characterized by an ability to colonize and spread throughout recipient habitats following dispersal (Lockwood et al., 2013). In cases where only a single or few individuals have become established, Allee effects can threaten persistence in a new location (Allee, 1951; Allendorf and Lundquist, 2003; Prentis et al., 2008). However, these factors can be offset by high rates of selfing though both within flower and between flower visits within a plant and matings between related plants, clonal propagation, or a combination, with the potential for inbreeding depression and subsequent purging of genetic load (Baker, 1955; Charlesworth and Charlesworth, 1987; Rambuda and Johnson, 2004). Alternatively, multiple introductions of plants from the same or different source populations can result in an establishment pathway that ameliorates the demographic and genetic constraints associated with an initially small colonizing cohort (Facon et al., 2006; Lombaert et al., 2010). Multiple introductions have been found to lead to greater population size and genetic diversity that can mitigate novel environmental constraints (Genton et al., 2005; Dlugosch and Parker, 2008; Wilson et al., 2009; Estoup and Guillemaud, 2010). A recent study has also demonstrated the role of the bridgehead process (where non-native populations act as a seed source for further non-native species expansion) and admixture between populations in helping to explain the global invasion of *Mimulus guttatus* (Vallejo-Marin et al., 2021).

The aim of this study was to examine the mating system *in situ* in perennial populations of the self-compatible *Mimulus guttatus* that occur along the invasion spectrum: *native* populations that have been evolving in their environments for millennia; *naturalized* populations that have established outside their native range but have not spread; and *invasive* populations that have dispersed beyond the point of initial introduction (Richardson et al., 2000). Studies of plant invasions that include comparisons among native, naturalized, and invasive populations are uncommon because naturalized populations are often cryptic and benign in the environment, and thus difficult to identify in the field (*but see* Muth and Pigliucci, 2006). We tested the following hypotheses: (1) Outcrossing rates and mean inbreeding coefficients differ among populations in three regions: native, naturalized, and invasive. If non-native populations of *M. guttatus* adhere to Baker’s Law, we predict that outcrossing rates will be lower, and inbreeding coefficients higher, in naturalized and invasive populations compared to native populations; (2) Inbreeding depression differs among populations in the three regions. We predict that inbreeding depression will be lower in populations that have lower outcrossing rates because they may have been established a sufficient length of time to have purged their genetic load, leading to an equilibrium state.

## Methods

### Study species

*Mimulus guttatus* (Phrymaceae; 2*n* = 2*x* = 28), or common monkeyflower, is a self-compatible mixed-mating species native to the West coast of North America, found from Mexico to Alaska (Dudash et al., 1997; Carr et al., 1997; Kelly and Arathi, 2003; Wu et al., 2008; Lowry et al., 2008). In its native range, *M. guttatus* populations can be found exhibiting either an annual (ephemeral water availability) or perennial life history (persistent water availability) (van Kleunen and Fischer, 2008; Lowry et al., 2008; Popovich and Lowry, 2020; Zell et al., 2023). *Mimulus guttatus* is pollinated by bees and other insects who readily visit multiple flowers on an individual. i.e., geitonogamy, suggesting limited gene flow via pollen dispersal (van Kleunen and Johnson, 2007). The seeds produced per fruit are often in the 100s and are small and can potentially be dispersed long distances by water and possibly wind (Grant, 1924; van Kleunen and Fischer, 2008).

Native populations of *M. guttatus* exhibit wide variation in outcrossing rates (Ritland and Ganders, 1987; Dudash and Carr, 1998). Transitions between outcrossing and selfing within the *Mimulus* genus (Fenster and Ritland, 1994) are likely controlled by polygenic inheritance (Fenster et al., 1995). The mode of self-fertilization in *M. guttatus* is largely competing selfing, which occurs concurrently with outcrossing through both within flower and geitonogamous self-pollination, as opposed to prior or delayed selfing (Lloyd and Schoen, 1992; Leclerc-Potvin and Ritland, 1994).

In addition to its sexual mating strategies of reproduction, *M. guttatus* is capable of forming clones via asexual reproduction, i.e., fragmentation and stolon production when water is persistent across years (Grant, 1924; Vickery, 1959; Truscott et al., 2006; Zimmer et al., 2022). *Mimulus guttatus* has become a model system in studies of evolutionary, developmental, and population genetics because of its broad phenotypic and genetic diversity (e.g., Dudash et al., 2005; Wu et al., 2008; Yuan, 2019).

### Study populations

For this study, we only sampled from *perennial native* populations because all known non-native populations are perennial. The eight native populations sampled, and their coordinates (**Table 1**) include three from Alaska (AKS1, AKS2, AKA), one from Washington (WA), three from Oregon (OR02, OR04, and OR06), and one from California (BB1). In prior work, we identified a few isolated populations in Eastern North America that have become *naturalized* and shown no detectable spread beyond their current locations (Murren et al., 2009 and Dudash pers. obs.). Little is known of the evolutionary history of these naturalized populations, but the two included in this study, Fly Creek, NY (FC) and Springfield, New Brunswick, Canada (NBS), are thought to have established over 50 years ago (Murren et al., 2009). In the United Kingdom (UK), *M. guttatus* is considered an *invasive* that was intentionally introduced as a horticultural species over 200 years ago (Truscott et al., 2008; van Kleunen and Fischer, 2008; Vallejo-Marin et al., 2021).

**Table 1:** Location and approximate population size of 8 *Mimulus guttatus* populations from the native region, two from the naturalized region, and three from the invasive region used for progeny array analyses.

Native region populations	Code	Latitude	Longitude	# of families used in progeny array (%with inferred maternal genotypes)	Approximate # individuals in population
Seward, AK (1)	AKS1	60.07021	-149.28102	19 (21%)	> 1000
Seward, AK (2)	AKS2	60.12142	-149.25660	20 (25%)	>200
Anchorage, AK	AKA	63.33945	-148.49102	11 (27%)	>1000
Shelton, WA	WA	47.23089	-123.08862	20 (15%)	>1000
Oswald State Park, OR	OR06	45.45797	-123.58105	21 (5%)	>1000
Haceta Head, OR	OR04	44.08163	-124.07605	20 (30%)	>500
Humbug Mt. State Park, OR	OR02	42.43072	-124.27879	8 (12.5%)	>500
Bodega Bay, CA	BB1	38.31701	-123.07117	20 (20%)	>1000
Naturalized region					
Fly Creek, NY	FC	42.44391	-74.58212	16 (44%)	>1000
Springfield, New Brunswick, Canada	NBS	45.41486	-65.49202	16 (6%)	>500
Invasive region					
Brampton, Norfolk, UK	BRA	52.7681	-1.27985	19 (100%)	>100
Dunblane, Perthshire, UK	DBL	56.18861	-3.96608	8 (100%)	>300
Houghton Lodge, Hampton, UK	HOU	51.09699	-1.5084	20 (100%)	>100

Each progeny array consisted of randomly chosen families in a population, and each family may or may not have been represented by a maternal individual along with eight progenies. If a family had an absent maternal individual, it was inferred from the progeny genotypes and allele frequencies by BORICE (Koelling et al., 2012).

### Sampling and genotyping for progeny array analysis

We conducted a primarily field-based progeny array analysis to estimate outcrossing rates (*t*), inbreeding coefficients (*F*), and relative fitness (inbreeding depression) of progeny for *M. guttatus* populations in the native, naturalized, and invasive ranges. This was completed in one season along the West coast of North America and the East coast as noted above (**Table 1**). The field route to survey the eight West coast native perennial populations was based on records obtained from colleagues (B. Blackman and D. Lowry, pers. comm.) and local contacts. For each of the 10 native and 2 naturalized populations visited, we randomly sampled seed and leaf tissue from 30-50 maternal families that were > 1 m apart to increase the chances of sampling multiple genotypes. Leaf tissue was immediately stored in silica gel until DNA extraction. For the three *M. guttatus* populations in the invasive region in the UK, we did not collect leaf tissue, but instead obtained field-collected seed from a colleague (M. Vallejo-Marin).

Seed representing progeny for all maternal families was grown in the University of Maryland (UMD) greenhouse and when seedlings were ~ 6 cm tall, leaf tissue was collected from eight progeny/maternal family and stored in silica gel. Up to thirty maternal families per population were randomly sown but fewer families were used in the progeny array analysis due to variable germination success and poor-quality DNA in some families (**Table 1**). This approach only captures the very early life history stages of the progeny while field collected tissue from maternal plants reflect selective pressures up to the adult stage. Furthermore, sampling was limited in the non-native regions (5 versus 10 native).

We extracted DNA from field-collected maternal leaf tissue (all populations except the three from the invasive region) and from leaf tissue collected from greenhouse-grown progeny originating from field-collected seed (all populations including the invasive populations). We used a modified CTAB protocol and an automated Autogen^®^ robotic DNA extractor (Doyle and Doyle, 1990). Genotyping methods and amplification protocol is provided in **Appendix 1**.

### Bayesian estimation of t and F using BORICE

We chose to use the software BORICE (Bayesian Outcrossing Rate and Inbreeding Coefficient Estimation; Koelling et al., 2012; http://www.python.org/) because of its ability to statistically infer maternal genotype when unknown and the Bayesian based program can calculate both outcrossing rates (*t*) and inbreeding coefficients (*F*) when sampling numerous maternal families with as few as 8 progeny per maternal plant. Maternal genotypes for the three UK populations had to be inferred along with some families from the native and naturalized populations when missing maternal genotype data (**Table 2**). This approach has been shown to be preferable to Ritland (1990) MLTR approach because of its ability to estimate mating system parameters with fewer progeny allowing larger maternal family sampling (Koelling et al., 2012). However, this approach also assumes that populations are in equilibrium, which can impact its reliability owing to its sampling design preferring more families and fewer progeny per maternal family to infer population level information. Details on BORICE joint estimation procedure can be found in **Appendix 2**.

**Table 2:** Outcrossing rates (*t*), inbreeding coefficients (*F*), and inbreeding depression (1-ω) in native and non-native populations of *M. guttatus*.

Native Population	*N* (# fams)	Unique MLGs	*P*	*N_a_* (range)	*H_o_* (SE)	*H_e_* (SE)	*t* (95% credible interval)	*F* (95% credible interval)	% missing data	Inbreeding depression (1-ω)
AKS1	177 (20)	118	9	2.73 (1-5)	0.17 (0.06)	0.18 (0.06)	0.91 (0.79, 0.99)	0.03 (0.00, 0.09)	12.2	0.37
AKS2	173 (20)	134	1 0	3.18 (1-4)	0.20 (0.06)	0.30 (0.08)	0.80 (0.70, 0.89)	0.06 (0.00, 0.12)	16.4	0.49
AKA	96 (11)	88	9	2.27 (1-3)	0.25 (0.06)	0.33 (0.07)	0.77 (0.65, 0.87)	0.06 (0.00, 0.18)	11.6	0.57
WA	176 (20)	169	1 0	1.94 (1-6)	0.24 (0.07)	0.37 (0.08)	0.81 (0.73, 0.88)	0.08 (0.03, 0.15)	15.2	0.26
OR06	188 (21)	135	9	2.46 (1-4)	0.16 (0.05)	0.25 (0.07)	0.74 (0.62, 0.85)	0.07 (0.00, 0.14)	17.2	0.57
OR04	174 (20)	122	9	2.64 (1-6)	0.17 (0.05)	0.26 (0.07)	0.70 (0.59, 0.81)	0.09 (0.03, 0.17)	14.9	0.54
OR02	71 (8)	47	7	2.18 (1-4)	0.15 (0.06)	0.15 (0.06)	0.90 (0.73, 1.00)	0.03 (0.00, 0.16)	12.0	0.44
BB1	176 (20)	167	9	3.70 (1-7)	0.26 (0.06)	0.36 (0.08)	0.74 (0.65, 0.82)	0.11 (0.03, 0.19)	9.0	0.30
Naturalized										
FC	136 (16)	42	6	1.70 (1-3)	0.02 (0.01)	0.16 (0.06)	0.63 (0.38, 0.82)	0.07 (0.00,0.17)	18.9	0.74
NBS	143 (16)	16	1	1.00 (1-2)	0.003 (0.002)	0.003 (0.002)	0.62 (0.14, 0.98)	0.19 (0.00, 0.67)	17.3	0.23
Invasive										
BRA	166 (19)	143	1 1	2.91 (0.25)	0.27 (0.06)	0.30 (0.06)	0.81 (0.71, 0.90)	0.03 (0.00, 0.08)	18.6	0.74
DBL	70 (8)	70	1 0	4.09 (1-8)	0.40 (0.07)	0.44 (0.07)	0.95 (0.89, 1.00)	0.01 (0.00, 0.06)	3.4	0.62
HOU	174 (20)	144	1 0	3.46 (1-6)	0.21 (0.06)	0.31 (0.07)	0.58 (0.49, 0.67)	0.12 (0.04, 0.21)	20.1	0.62

*N* number of individuals in progeny array (number of families in parentheses), maternal and progeny; the unique MLGs is the number of *N* that represent a unique multilocus genotype in the progeny array; *P* number of polymorphic loci in each population; *Na* average number of alleles per locus; *H_o_* observed heterozygosity; *H_e_* unbiased heterozygosity were calculated with microsatellite data using GENALEX (Peakall and Smouse, 2006); *t* outcrossing rate; *F* inbreeding coefficient calculated using BORICE (Koelling et al., 2012). Percent missing genotypes, across maternal individuals and progeny, for each population. BORICE infers missing maternal genotypes, and ignores missing genotypes in progeny; *Inbreeding depression* 1- {(2 x t x F)/[(1 - t)(1 – F)]} (Ritland, 1990).

We used a chain of 100,000 steps with a burn-in of 10,000 steps. Once the posterior *t* and *F* were estimated, we calculated Ritland (1990) estimator for relative fitness of selfed progeny (ω) to characterize inbreeding depression (1- ω) in each population. Assuming *F* is constant across generations, relative fitness is found using:


ω=2×t×F/[(1−t)(1−F)]

and inbreeding depression is 1-*ω*. Because inbreeding depression is dependent only on *t* and *F*, understanding different outcomes regarding the selection against selfed individuals is straightforward. For example, if both *t* and *F* in a population are low, inbreeding depression could be relatively high. This occurs because while many selfed progeny are produced (low *t*), few survive to adulthood (low *F* among maternal individuals). Alternatively, if *t* is low and *F* is high, the measure of inbreeding depression could be low because the high number of offspring produced by selfing survive and are represented in the next maternal individuals, as evidenced by the high *F*. Additionally, when outcrossing rates are high, the estimate of inbreeding depression will generally be low regardless of the magnitude of *F*, because when selfed progeny are produced they result in an opportunity to purge genetic load through selection eliminating deleterious recessive alleles. Finally, if there is high inbreeding depression in a selfing population this implies that purging is still ongoing and following many generations of selfing purging should further eliminate deleterious alleles and then we would expect lower inbreeding depression and higher inbreeding coefficients in the population.

### Statistical analysis

We used Welch’s t-tests (R Core Team, 2016), which do not require the assumption of equal variances between unpaired samples, to examine pairwise differences between *M. guttatus* regions (native, naturalized, and invasive) regarding *t*, *F*, and inbreeding depression (1- *ω*). All mating system and genetic diversity parameters comparisons were based on family averages for each study population’s estimates from one season.

## Results

For the 13 populations in the progeny arrays, we utilized 11 codominant markers (**Table 3**). These included five microsatellite loci previously used to genotype North American and British *M. guttatus* populations (Kelly and Willis, 1998; Vallejo-Marin and Lye, 2013), and six markers revealing length polymorphisms in introns of single-copy nuclear genes in *M. guttatus* (Fishman and Willis, 2005; Lowry et al., 2008; Vallejo-Marin and Lye, 2013; Zimmer et al., 2022). These intron length polymorphisms, or MgSTS (*M. guttatus* sequence-tagged sites) were found to be suitable for genotyping based on a selection strategy of Vallejo-Marin et al. (2011). These markers are variable in samples of *M. guttatus*, its close relative, the tetraploid *M. luteus*, and the triploid hybrid produced by them, *M. x robertsii* Silverside, and are suitable for multiplexing (Vallejo-Marin and Lye, 2013).

**Table 3:** Diversity of eleven molecular markers used in the study, across 13 native and non-native populations of *M. guttatus*.

Locus	Approx. size range (bp)	Total no. of alleles	H_o_	H_e_
AAT217	177-195	6	0.12	0.67
AAT225	113-127	14	0.07	0.50
AAT230	179-210	26	0.32	0.88
AAT240	94-106	10	0.18	0.76
AAT278	127-135	3	0.04	0.12
MgSTS84	209-231	10	0.21	0.66
MgSTS234	272-321	12	0.22	0.69
MgSTS321	290-303	12	0.15	0.68
MgSTS430	238-269	13	0.25	0.65
MgSTS681	338-366	8	0.31	0.73
MgSTS685	242-251	15	0.23	0.84
Average		11.73	0.19	0.65
SD		5.92	0.03	0.06

AAT markers are microsatellites, and MgSTS are intron-based polymorphic markers designed for *Mimulus guttatus* (Kelly and Willis, 1998).

All markers utilized were polymorphic, with between three and 26 alleles (**Table 3**). There was evidence for the presence of null alleles in the form of ‘impossible genotypes’ reported by BORICE. A total of 10 impossible genotypes for loci AAT230, AAT278, and MgSTS84 were reported in five populations (AKA, OR02, BB1, FC, and HOU) (Zimmer et al., 2022). Therefore, while null alleles were allowed in the final analysis of all populations, a random sample of three populations (AKA, OR04, and HOU) was run without allowing for null alleles and the results for *t* and *F* were not statistically different from the models that allowed for nulls (t-test, *P* > 0.05). The naturalized East coast FC population had the greatest number of private alleles (Zimmer et al., 2022).and the lowest number of unique MLGs (**Table 2**). Additionally, the percent of missing data across this study ranged from 3-20% (**Table 2**).

Family level averages of outcrossing rates (*t*), inbreeding coefficients (*F*), and inbreeding depression (1-ω) estimates were calculated to provide population level estimates for the native and non-native populations of *M. guttatus* in **Table 2**. Please note that there is much overlap among population mating system estimates when examining the 95% credible intervals, thus we will be discussing trends along with significant differences below.

### Outcrossing rates

Population level outcrossing rate estimates (*t*) in the native region on average were similar to those in the invasive region of *M. guttatus*, (native mean = 0.80 (SE ± 0.03), *n* = 8 populations; invasive mean = 0.78 (SE ± 0.11), *n* = 3 populations; t score = 0.15, *P* = 0.90). However, one population from the invasive region (HOU) had the lowest outcrossing rate recorded among all populations sampled, *t* = 0.58 while another had the highest *t* recorded (DBL, *t* = 0.95). Contrastingly, the mean *t* for the two naturalized populations was significantly lower than that in the native region (naturalized mean = 0.63 (SE ± 0.01), *n* = 2 populations; native mean = 0.80 (SE ± 0.03), *n* = 8 populations, t score = 3.63, *P* < 0.01). The mean estimate of *t* in the naturalized region (*t* = 0.63, SE ± 0.01) was also lower than the invasive region (*t* = 0.78, SE ± 0.11), but not significant (t score = 1.11, *P* = 0.35; **Table 2**).

### Inbreeding coefficients

The mean estimate of inbreeding coefficients (*F*) was also similar among the native and invasive regions (native mean = 0.07 (SE ± 0.01); invasive mean = 0.05 (SE ± 0.03); t score = 0.52, *P* = 0.62). The mean *F* in the two naturalized populations trended higher than *F* estimates in the native and invasive regions (naturalized mean = 0.13 (SE ± 0.06); vs. native, t score = 2.03, *P* = 0.08; vs. invasive, t score = 1.23, *P* = 0.31) but were not significant (**Table 2**).

### Inbreeding depression

Inbreeding depression (1 – *ω*, where *ω* = the relative fitness of progeny produced by selfing) was greatest in the invasive region, and significantly greater than that found in the native region (native mean = 0.44 (SE ± 0.04); invasive mean = 0.66 (SE ± 0.04); t score = 2.87, *P* = 0.02). The mean level of inbreeding depression in the naturalized populations was intermediate between native and invasive levels (naturalized mean = 0.49 (SE ± 0.26); vs. native mean = 0.44 (SE ± 0.04), t score = 0.31, *P* = 0.76; vs. invasive, t score = 0.88, *P* = 0.44, but there was a large amount of variation between estimates of the two naturalized populations, FC (0.74) and NBS (0.23), which was not significantly different **Table 2**).

## Discussion

The evolution of mating systems in colonizing plants has been a primary focus in ecology for decades and is likely to be case dependent, with demographic and stochastic variables playing consequential roles in determining the extent of selfing versus outcrossing in incipient populations (Baker, 1967; Cheptou, 2012; Pannell et al., 2015). This is the first study that we aware of to utilize BORICE to compare *in situ* outcrossing rates (*t*) and mean inbreeding coefficients (*F*) of maternal individuals between native populations of *M. guttatus* and two separate categories of non-native populations, naturalized and invasive. Our data revealed that overall native populations exhibited a relatively high outcrossing rates and lower inbreeding coefficient across the 8 populations as would be expected. However, inbreeding depression was quite variable with four native populations above or equal to 0.5 and the other four native populations below 0.5 suggesting different suites of factors likely influenced their mating system evolution. In contrast the three UK non-native populations differed in their reliance on selfing to persist in their respective locations demonstrated by their variable outcrossing rates. Only one naturalized populations (NBS) in Eastern North America, demonstrated an outcrossing rate greater than 0.5 and inbreeding depression below 0.5 as predicted from transmission bias theory (e.g., Fisher, 1941) compared to four populations in the native region that also demonstrated this pattern. The remaining two populations in the invasive UK region and the one naturalized population on the east coast had relatively high outcrossing rates, low adult inbreeding depression coefficients, and high inbreeding depression suggesting ongoing purging in these populations along with some populations in the native region. Below we discuss probable scenarios and limitations of our study including the limited sampling design in the non-native compared to the native region that may help explain the variable estimates of *t*, *F* and inbreeding depression found in these populations. We also explore how different mating system approaches and parameters may be limited in their ability to predict the maintenance of current and future mating systems in these populations

Outcrossing rate variation in both native and non-native *M. guttatus* populations suggested the persistence of mixed-mating system in these populations (**Table 2**). The prediction that we would see a positive relationship between outcrossing rate and inbreeding depression was weakly supported in three of the eight native populations, which are most likely to be in genetic equilibrium owing to their long-term persistence in the native range. We found little support for our prediction that non-native populations of *M. guttatus* would adhere to Baker’s Law, such that outcrossing rates would be lower and inbreeding coefficients higher in naturalized and invasive populations compared to native populations with only the naturalized NBS populations exhibiting relatively low *t* and high *F* (**Table 2**). In addition, the averages for *t* and *F* in the two UK populations (BRA, DBL) in the invasive region were nearly identical to that in the native region and differed from the UK HOU, which appeared to support Baker’s Law. The population estimate outcrossing rates among the eight native *M. guttatus* populations sampled of, 0.70-0.91 (even with their large 95% credible interval; **Table 2**), was approximately within the range of outcrossing rates found in past studies using MLTR (0.41-0.76, Ritland and Ganders, 1987; 0.68-0.80, Dudash and Ritland, 1991). This general agreement in outcrossing rates supports the population estimates found by BORICE (Koelling et al., 2012) here compared to MTLR prior estimates of Dudash and Ritland (1991) even with their contrasting approaches, sampling, and inherent assumptions.

Inbreeding coefficients in the native region were generally low, as would be expected with the outcrossing rates detected (**Table 2**). The estimates of *t* and *F* for two of the three populations from the UK, where *M. guttatus* is considered an invasive, fell within the ranges of *t* and *F* for the native region (**Table 2**). Our results support the idea that there can be variation in outcrossing rates among populations of the same species independent of how they are categorized (native, naturalized, or invasive) and likely in part because each population has its own unique past. An extensive survey of outcrossing rates of 105 plant flowering species represented by a minimum of 3 populations each, demonstrated great variation in outcrossing rates and warned about extrapolating the results of one population to the whole species (Whitehead et al., 2018). Dart et al. (2012) also documented three modal stable mixed mating systems among 40 populations across the range of *Camissoniopsis cheiranthifolia* that varied from predominately outcrossing with self-incompatible individuals with large flowers to moderate selfing populations with individuals with large self-compatible flowers to higher selfing populations associated of individuals with smaller autogamous flowers. Overall, our work and these studies suggest that the variation in mating systems observed is likely context dependent and a result of both environmental and genetic factors along with where a particular population lies along a continuum within its native range, at its edge, if and how long it has been naturalized, and its likelihood of becoming invasive.

It has been widely documented that invasive *M. guttatus* populations in the UK are the unintended consequence of the horticulture trade between the US and Europe over 200 years ago (Preston et al., 2002; Truscott et al., 2008; van Kleunen and Fischer, 2008; Puzey and Vallejo-Marín, 2014; Vallejo-Marin et al., 2021). By repeatedly introducing propagules to non-native regions (intentionally as a horticultural species, in the case of *M. guttatus*), the likelihood of forming natural populations with greater densities and genotypic diversity increases (Kalisz et al., 2004; Eppley and Pannell, 2007; Friedman and Barrett, 2008). A positive correlation between outcrossing rates and plant density has frequently been shown in herbaceous plant species (Karron et al., 1995; Herlihy and Eckert, 2004; Brunet and Sweet, 2006). For this study, we estimated population size by the number of adult plants present at each of the 13 locations when we collected maternal family seed and found no clear relationship between estimated population size and outcrossing rate, inbreeding coefficients and inbreeding depression perhaps in part because of the overall size similarities of the populations sampled (i.e., readily detected) for this study (**Table 1**).

Many ornamentals were introduced into Europe (Reichard and White, 2001; Mack, 2003; Thuiller et al., 2005); because of traits associated with reproductive fitness, such as floral size and biomass. These same traits are also thought to increase the likelihood that escaped plants will successfully establish outside of their native range (Mack, 2000; Kowarik, 2003). It is an open question as to whether *M. guttatus* populations in introduced regions display differences in various fitness traits that may facilitate establishment and invasion. For example, one study compared floral traits, along with sexual and vegetative reproduction, between invasive versus native populations of *M. guttatus* and found no significant differences (van Kleunen and Fischer, 2008). However, a companion greenhouse study using these same populations, found evidence for a significant increase in floral size (floral width *x* floral length) in invasive populations compared to native populations (Berg, 2018) and a significant reduction in floral size of the naturalized populations. Interestingly, there was no difference in stigma-anther separation among the populations across native, naturalized, and invasive populations in this study even though floral size varied (Berg, 2018) suggesting uniform selection against within floral self-pollination. Because increasing floral size traits have been associated with higher outcrossing rates in *M. guttatus* (Ritland and Ritland, 1989), more research is needed to evaluate the role of enhanced sex allocation to increasing floral size in the mating systems of non-native populations.

In one UK population (HOU), and in one naturalized population on the East coast (NBS), outcrossing rates were lower and the mean inbreeding coefficient among maternal individuals tended to be greater compared to native populations. In the naturalized populations, the lower outcrossing rates can be explained by the lack of genotypic diversity in each population for total number of alleles per loci and average number of alleles per loci such that outcrossing events could not be readily detected even when they occurred (Zimmer et al., 2022). The observed heterozygosity from limited microsatellite data in the naturalized FC and NBS populations was much lower compared to the 11 other populations (**Table 2**) suggesting that asexual clonal propagation, geitonogamy, and fixation of alleles were important characteristics of these naturalized East coast *M. guttatus* populations (Zimmer et al., 2022). When examining the expected heterozygosity (He) versus observed heterozygosity (Ho) of the naturalized populations the FC population He is much higher than the Ho while both Ho and He are low in NBS population. This suggests there are more alleles in FC than NBS and the reason for the low Ho is different (confirmed in Zimmer et al., 2022). In NBS an outcrossing event cannot be as easily detected owing to a lack of genetic variation while this is not necessarily the case for the FC population. Furthermore, the number of private alleles detected for FC (n=5) compared to NBS (n=0) supports this line of reasoning (Zimmer et al., 2022). Another parameter that provided further insight was the proportion of unique multilocus genotypes (MLGs) determined with *poppr* (Zimmer et al., 2022) which produced outcrossing estimates lower in the naturalized east coast populations compared to the native and invasive UK populations. On the East coast FC and NBS, the proportion of unique MLGs of the total number of individuals sampled, maternal and progeny, was only 31% and 11%, respectively. These proportions are low compared to an average 82% (SD = 0.13) across the remaining 11 populations in the study. Pantoja et al. (2017) provides further evidence for great differences in genetic variation and clonal reproduction among 14 populations of *M. guttatus* in the non-native invasive UK range when compared to 10 native populations. Their results demonstrate a great breadth in how non-native populations (invasive and naturalized) establish through sexual versus asexual reproduction as well as hints to historical routes of introduction into the UK. Together these studies highlight the potential role of uniparental (asexual through clonality) reproduction in non-native population persistence and their ability to spread outside their native range.

The lack of genotypic and genetic diversity in the progeny arrays of the East coast naturalized populations (observed heterozygosity in FC was 0.02, and effectively zero in NBS; **Table 2**) results in an inability to distinguish whether any particular offspring was the result of selfing, because the progeny genotypes are identical to the maternal genotype, or rather of outcrossing within a genet (geitonogamy), because many of the maternal genotypes are identical to one another. In these cases, the Bayesian posterior probability of *t* will approach 0.5 because half of the offspring will be deemed the product of a selfing event, and the other half of the product of outcrossing between parents that share the same genotype. Evidence for lack of confidence in determining whether progenies were produced via selfing or outcrossing in the two naturalized populations can be seen in the high variability surrounding the mean posterior estimate of *t* in FC and NBS (**Table 2**). This lack of genotypic and genetic diversity in the East coast populations could be the result of limited opportunities for colonization, a recent bottleneck (Tsutsui et al., 2000; Frankham, 2005; Prentis et al., 2008), a dominating role of genetic drift over new mutations (Crooks et al., 1999; Eckert et al., 1996), or their dependence on both sexual and asexual reproduction (Pantoja et al., 2017). Why these two naturalized populations with similar outcrossing rates, low observed heterozygosity, but contrasted markedly in inbreeding depression have not expanded is unclear and alludes to how one year’s data can be inadequate to understand the history of any population over time. Both populations were discovered in the early 2000s (Dudash pers obs.) and their ongoing persistence could be in part due to their dependence on water availability owing to their perennial growth form. Another explanation is that we did not search in a wide enough area to detect the establishment of other populations in their respective regions of the east coast.

In the invasive UK populations, the resulting inability to distinguish selfing events from outcrossing within clones and the lower outcrossing rate in the HOU population can be attributed with more confidence to actual selfing events. This is because at least in part to nearly all maternal individuals (93%) exhibiting a unique MLG, making exclusion of paternity via an outcrossing event more straightforward compared to the naturalized east coast populations. Additionally, the UK HOU population appears to be on a different evolutionary trajectory compared to the UK DBL population because it exhibited a lower outcrossing rate and higher inbreeding coefficient than the DBL population with the highest outcrossing rate and lowest inbreeding coefficient of the invasive populations even though they were found to be most similar using Nei’s genetic distance (Zimmer et al., 2022; see Figure 3) suggesting they may be at different places along the continuum of invasion although no relative establishment dates are known.

The contrast in outcrossing rates in the UK invasive region between the lower *t* found in the HOU population and the other two populations with relatively higher *t* values (BRA and DBL) is interesting and could be associated with any number of factors, including smaller effective population size in the HOU population or differences in environmental factors (Schemske and Lande, 1985; Ellstrand and Elam, 1993; van Treuren et al., 1993). Another possibility is that HOU is the result of a bridgehead effect, a phenomenon in which the source of a non-native population or invasive population is not from the native region, but rather from a distant non-native population (Lombaert et al., 2010). Recently Vallejo-Marin et al. (2021) have demonstrated the high likelihood of non-native populations in the UK contributing to the establishment of *M. guttatus* populations globally. Taking this idea one step further, we can imagine a scenario where one of the naturalized east coast populations may have contributed propagules to the establishment of the invasive UK HOU population. A genetic comparison based on Nei’s genetic distance (Nei, 1978) from a companion study of the same populations studied here (Zimmer et al., 2022) showed that the UK HOU and DBL populations are closely related but more distantly related to the UK BRA population and could be evidence of a UK population providing the source propagules for another UK population. Additionally, the naturalized NBS population could also be the result of introductions from the invasive UK populations (Zimmer et al., 2022). Collectively these *M. guttatus* studies provide evidence for potential bridgehead effects as a pathway to invasion for non-native plant populations and could serve as an interesting model to investigate this phenomenon in other invasive plant species in the future.

Now turning to inbreeding depression, six (five native and one non-native naturalized) of the thirteen *M. guttatus* populations demonstrated levels of inbreeding depression below 0.5, the theoretical threshold where an allele causing selfing should potentially be able to spread through an outcrossing population and there has been a sufficient length of time to purge the major deleterious alleles from the population. (Fisher, 1941; Lande and Schemske, 1985; Ritland, 1990) In agreement with our prediction, inbreeding depression levels did appear to depend on a population’s geographic region with inbreeding depression levels greater in four out of the five non-native populations compared to the native populations suggesting the maintenance of an outcrossing mating system. In four native populations we found *t* ≥ 0.8, *F* was relatively lower, and all inbreeding depression estimates were below 0.5, supporting the potential evolution of selfing by the 3/2 transmission bias over outcrossing (e.g., Fisher, 1941). Subsequently, there are various scenarios that could explain this one year’s parameter estimates and their potential implications to whether selfing is expected to evolve or is still actively being purged from a population promoting outcrossing.

Recall that we calculated an *in-situ* level of inbreeding depression for each population from field collected seed that germinated in a greenhouse and collected as seedlings such that both very early and later life history stages were not captured likely leading to underestimates (e.g., Husband and Schemske, 1996; Byers and Waller, 1999). Additionally, the progeny collected at the seedling stage in the greenhouse likely minimizes the impact of inbreeding depression compared to the tissue collected from surviving maternal parents in the field. Furthermore, *M. guttatus* is capable of producing 100s of seeds per flower and numerous flowers per plant (e.g., Dudash and Ritland, 1991) thus 8 progeny per maternal family is not ideal to capture a robust estimate of individual adult plant fitness in the field. Finally, we examined only one generation to calculate inbreeding depression that was measured by using *t* as an estimate of the proportion of selfed progeny produced and mean maternal *F* as an estimate of the number of those selfed individuals that survive to adulthood with the assumption that all populations were in equilibrium, which was not tested and could also be contributing to the large variation in our estimates (Ritland, 1990).

It has been repeatedly shown that inbreeding depression can be intense in early life history stages (e.g., Husband and Schemske, 1996), and later life history stages (e.g., Dudash et al., 1997), thus deleterious alleles can be purged throughout a plants life history and inbreeding depression is likely dependent on variation in the maternal family mating history (Dudash and Carr, 1998), particularly when negative density-dependent interactions occur in populations (Mitchell-Olds and Waller, 1985). Results from studies focusing on *M. guttatus* have concluded that inbreeding depression impacts pollen viability (Willis, 1993), and pollen and ovule production (Carr and Dudash, 1995; Carr et al., 1997; Dudash and Carr, 1998) and is expressed throughout the later life history stages as well (e.g., total flower production, adult above-ground biomass; Dudash et al., 1997). There is also evidence of differential purging of genetic load in *M. guttatus*, among maternal families, when quantifying later life history traits (Dudash et al., 1997; Dudash and Carr, 1998). A companion greenhouse study investigated the magnitude of inbreeding depression across early and later life history stages among these same populations and the results supports prior work in *M. guttatus* with once again finding that self-progeny accumulated significantly less above-ground biomass than outcrossed progeny (Berg, 2018).

Another explanation of why we observed inconsistency among outcrossing rates, inbreeding depression, and the inbreeding coefficient may be because some populations had not met equilibrium assumptions (Ritland, 1990). This is more likely in our study because we sampled each population only once, thus we did not have multiple generations of inbreeding coefficients that would have added rigor when calculating these mating system parameters. Ritland (1990) demonstrated that when equilibrium assumptions were not met large variances in inbreeding depression were detected. Furthermore, the inbreeding depression estimates often did not correlate with selfing rate and there was often a positive bias in the estimate leading to false negative correlations between inbreeding depression and selfing rates. Our study supports Ritland (1990) findings because we did not find a consistent relationship between inbreeding depression and outcrossing rate estimates using the one-year snapshot approach, warranting further investigation.

The only East coast naturalized non-native population that fell below the inbreeding depression threshold of 0.5 was the clonal NBS population, which consisted of only a few unique multilocus genotypes (MLGs; **Table 2**). All loci were also monomorphic for a single allele except for locus AAT225, which had a second, rare allele (frequency of allele “97” = 0.99, frequency of allele “103” = 0.01; Zimmer et al., 2022 see **Table 3**). It is difficult to know whether the genetic diversity described in NBS is the result of an origin in which a single or few genotypes were introduced and through subsequent selfing and asexual vegetative propagation resulted in a genotypically homogenized monoculture at this location. It is also possible that multiple introductions occurred at this location, and through selection, drift, or a combination of the two, and concurrent high rates of selfing resulted in the genetic pattern observed. There has been much discussion about the ability of invasive plant populations to purge their genetic load and escape the detrimental effects of inbreeding depression resulting in populations where selfing is evident and outcrossing less pronounced (Byers and Waller, 1999; Dudash and Fenster, 2000; Frankham, 2005) and whether in particular instances some genetic load may be beneficial for establishment under novel environmental conditions for long term population persistence (Bazin et al., 2014). Whether repeated introductions of novel individuals into a population slows down the purging process through selfing or mating between relatives, could perhaps explain the divergent results in the two East coast naturalized populations that had similar outcrossing rates and divergent inbreeding depression estimates for the one year of data presented in this study

### Conclusion

In this one-year study we have shown that mating system estimates in native, naturalized, and invasive plant populations of *M. guttatus* overall support the existence of mixed-mating populations in both the native and non-native regions. There appears to be a relationship between outcrossing rate, population location and persistence history in a particular region. Additionally, we observed variation in estimates of outcrossing rate, inbreeding coefficient, and inbreeding depression. This is in part because of the methodology utilized to estimate the mating system parameters and their inherent sampling design and assumptions. Nevertheless, we have provided evidence for trends supporting the predicted weak relationship between higher outcrossing rates and lower inbreeding depression in the native range and both high rates of outcrossing (with high inbreeding depression) and lower rates of outcrossing (with lower inbreeding depression) in non-native populations of a single species. These results might be due at least in part to the mid-range heterozygosity levels found across the native and invasive study populations but not the naturalized populations. We have also found support for the bridgehead process, which can be thought of as a stepping stone, where one established invasive population provides seed for the establishment of another non-native population. Where a specific population at any point in time we investigate falls along a continuum of their historical persistence in a location is likely variable and dependent on many factors including both sexual and asexual reproduction for these perennial *M. guttatus* populations.

## Data Availability

The datasets presented in this study can be found in online repositories. The names of the repository/repositories and accession number(s) can be found below: https://doi.org/10.5061/dryad.cvdncjt7c, 0000.
