# Supplementary material for: Progeny array analysis to estimate outcrossing rates, inbreeding coefficients, and inbreeding depression among native, naturalized, and invasive populations of Mimulus guttatus (Phrymaceae)
Source: Front Plant Sci. 2024 Nov 21;15:1411868. doi: 10.3389/fpls.2024.1411868 (PMC11617154; doi:10.3389/fpls.2024.1411868)
Supplement: Supplementary file 1 [file DataSheet1.pdf]

Appendix 1. DNA extraction process.

DNAs were amplified for the 11 loci in sets of two multiplexed reactions using a 2 x Qiagen Type-It Microsatellite PCR kit (Qiagen, California, USA), 2 µM of each of the fluorescent forward primers labeled with either FAM or HEX dyes and 2 µM of each reverse primer and 5-50 ng of template DNA. PCR cycles consisted of a denaturing step of 5 min at 95 °C, followed by 30 cycles of 95 °C for 30 s, 55 °C for 180 s and 72 °C for 30 s and a final elongation step of 30 min at 60 °C. We examined success of the PCR amplifications in a 1.5% agarose 1x sodium hydroxide-boric acid buffer electrophoresis gel (Brody & Kern 2004). PCR products were diluted in nuclease-free water (dilutions ranged from 1:10 to 1:50), and one µL of each dilution was added to 9 µL of HiDi formamide with 1 µL ROX standard (DeWoody et al. 2004). Samples were heated to 95 °C for six minutes, cooled to 4 °C for six minutes, and loaded onto an ABI 3730xl automated capillary sequencer with a 50 cm, 96 channel array containing POP-7 polymer for fragment analysis at the Laboratories of Analytical Biology (LAB) of the Smithsonian National Museum of Natural History.

We performed allele binning and analyzed raw peak sizes from fluorescent fragment profiles using GeneMapper v5.0 software (Applied Biosystems), which allows calling of multiple peaks per locus. Maternal or progeny that failed to amplify were run a second time. Those that failed following the second run of PCR were left out of the analysis. A random sample of 10% of individuals that were successfully genotyped was then re-assayed and re-scored to check consistency.

Literature Cited

Brody JR, Kern SE (2004) Sodium boric acid: a Tris-free, cooler conductive medium for DNA electrophoresis. Biotechniques 36:1–2.

DeWoody JA, Schupp, J, Kenefic, L, Busch, J, Murfitt, L, Keim P (2004) Universal method for producing ROX-labeled size standards suitable for automated genotyping. Biotechniques, *37*, 348-352.
