# Supplementary material for: Progeny array analysis to estimate outcrossing rates, inbreeding coefficients, and inbreeding depression among native, naturalized, and invasive populations of Mimulus guttatus (Phrymaceae)
Source: Front Plant Sci. 2024 Nov 21;15:1411868. doi: 10.3389/fpls.2024.1411868 (PMC11617154; doi:10.3389/fpls.2024.1411868)
Supplement: Supplementary file 2 [file DataSheet2.pdf]

Appendix 2: BORICE Joint Estimation Procedure.

The likelihood for family *k*, l_k, is:

$$lk = \Pr[M_k]\prod_{i=1}^{n_k}(tP_{out}\,[A_{ik}|M_k] + (1-t)P_{in}[A_{ik}|M_k]) \qquad (\text{Eqn. }1)$$

where M_k is the vector of genotypes for maternal individual *k*; A_{ik} is the vector of genotypes for progeny *i* of maternal individual *k*; and n_k is the number of individuals in family *k*. The probability of M_k depends on population allele frequency and the latent (i.e. unobserved) variable, C_k, which is the inbreeding history of each maternal individual. In BORICE, C_k values equal the number of generations of selfing in the ancestry of maternal individual and are used to determine the inbreeding coefficients for maternal individuals (either absent or present) in the progeny arrays. The relationship between C_k and *F* is:

$$F = 1 - \left(\frac{1}{2}\right)^{C_k} \qquad (\text{Eqn }2)$$

For example, when C_k = 0, then the individual is outbred and *F* = 0. When C_k = 1, the individual is considered a selfed progeny of maternal individual with C_k = 0, and therefore *F* = ½. When C_k = 2, the individual is a selfed progeny of a maternal individual with C_k = 1 and *F* = ¾, and so on. The C_k values are integers ranging from 0 to 6. Values larger than 6 result in a negligible difference in *F* compared to C_k = 6, and therefore BORICE pools these individuals with those with a C_k = 6. With *F*, the maternal genotype probability at a given locus *x* is dependent on allele frequencies and given by standard formulas (Hartl and Clark 1989):

$$Prob[A_iA_j] = 2(1-F)q_{xi}q_{xj} \text{ for } A_i \neq A_j \text{(heterozygote)} \quad \text{(Eqn 3a) or}$$

$$Prob[A_iA_i] = (1-F)q_{xi}^2 + Fq_{xi} \text{ for homozygotes} \qquad \text{(Eqn 3b)}$$

where q_{xi} is the allele frequency at locus *x*. $\Pr[M_k]$, $P_{out}[A_{ik}|M_k]$, and $P_{in}[A_{ik}|M_k]$ are products over loci, and loci are assumed to be unlinked.

To estimate the parameters included in the likelihood function, BORICE uses Markov Chain Monte Carlo with a Metropolis-Hastings algorithm (Metropolis et al. 1953). The outcrossing rate *t* and the allele frequencies are standard parameters estimated from the observed data, while C_k and all unknown maternal genotypes are latent variables. BORICE assumes a uniform prior density for both *t* and the allele frequencies, and an iteration of the chain has four stages: 1) Propose and accept/reject adjustment to *t*; 2) Propose and accept/reject adjustment to q_{xi} for each locus *x* in series; 3) Propose and accept/reject new value of C_k with each maternal plant *k* considered in series; 4) Propose and accept/reject a new genotype for a random locus of maternal genotype M_k within each family *k* considered in series.

The proposed value *t'* for each iteration of the chain is the current value *t* summed with a small increment adjustment. The default range of incremental adjustment in BORICE is uniform between -0.05 and 0.05. The proposal ratio (R) is:

$$R = \frac{Prob[Data|t']}{Prob[Data|t]} \quad \text{(Eqn 4)}$$

If R > 1, then the proposed adjustment is accepted. If R < 1, a uniform random number *u* is drawn and if *u* < R the adjustment then *t'* is accepted.

For allele frequencies, a score is updated and tracked, y_{xi}, corresponding to each allele *i* at each locus *x*. Updates to y_{xi} are made using the same methods as for updates to *t*, and is based on previous work on proportion variables in phylogenetics (Lewis et al. 2010):

$$R = \frac{Prob[Data|y'_{xi}]}{Prob[Data|y_{xi}]}\left(e^{y_{xi}-y'_{xi}}\right) \quad \text{(Eqn 5)}$$

For the latent variables (i.e. C_k values and missing maternal genotypes), proposed values are sampled probabilistically based on current values of t and allele frequencies. The proposed value of C_k of maternal plant k is sampled from a geometric distribution: Prob[C'_k = 0] = t, Prob[C'_k = 1] = (1-t)t, Prob[C'_k = 2] = $(1-t)^2t$, Prob[C'_k = 3] = $(1-t)^3t$,… Prob[C'_k=6] = 1 – $\sum_{i=0}^{5} \text{Prob}[C'k\ = i]$. The proposal ratio for C'_k values is family specific likelihood (i.e. changes to C_k affect only one family):

$$R = \frac{Prob[C'_k|M_k]}{Prob[C_k|M_k]} \quad \text{(Eqn 6)}$$

Imputed maternal genotypes are sampled from the probability distribution derived from current allele frequencies and C_k values (Eqn 3). The proposal ratio, like that of C_k is family specific and given by:

$$R = \prod_{i=1}^{n_k}\left(\frac{tP_{out}[A_{ik}|M'_k] + (1-t)P_{in}[A_{ik}|M'_k]}{tP_{out}[A_{ik}|M_k] + (1-t)P_{in}[A_{ik}|M_{k]}}\right) \quad \text{(Eqn 7)}$$

Prior to analysis, 10 impossible genotypes for loci AAT230, AAT278, and MgSTS84 were reported by BORICE (i.e. genotypes in progeny that do not contain at least one maternal allele). Therefore, we followed advice by Koelling et al. (2012) and allowed null alleles in the model to calculate family likelihoods, l_k.

Literature Cited

Hartl DL, Clark AG (1989) Principles of Population Genetics 3rd ed. Sinauer Associates. USA.

Koelling VA, Monnahan PJ, Kelly JK (2012) A Bayesian method for the joint estimation of outcrossing rate and inbreeding depression. Heredity 109:393–400.

Lewis PO, Holder MT, Swofford DL (2010) Phycas User Manual. Version 1.2.0.

Metropolis N, Rosenbluth AW, Rosenbluth MN, Teller AH, Teller E (1953) Equation of state calculations by fast computing machines. The Journal of Chemical Physics 21:1087–1092.
